# Liver Retraction Using n-Butyl-2-Cyanoacrylate (NBCA) Glue during Laparoscopic Splenectomy and Azygoportal Disconnection in Cirrhotic Patients

**DOI:** 10.1155/2018/3064046

**Published:** 2018-08-19

**Authors:** Du Kong, Wei Wang, Gang Du, Binyao Shi, Zhengchen Jiang, Bin Jin

**Affiliations:** ^1^Department of General Surgery, Qilu Hospital of Shan Dong University, Jinan 250012, China; ^2^Department of Biochemistry and Molecular Biology, School of Medicine, Shandong University, Jinan 250012, China

## Abstract

**Background:**

Although liver retraction using n-butyl-2-cyanoacrylate (NBCA) glue has been applied to laparoscopic upper abdominal surgery in noncirrhotic patients, there is still no consensus on its safety and feasibility for cirrhotic patients. In this study, we aimed to investigate the safety and effectiveness of liver retraction using NBCA glue during laparoscopic splenectomy and azygoportal disconnection (LSD) for gastroesophageal varices and hypersplenism secondary to liver cirrhosis and portal hypertension.

**Methods:**

Thirty-nine gastroesophageal varices and hypersplenism secondary to liver cirrhosis and portal hypertension patients were included in our study. We performed LSD in the presence of NBCA glue (n = 22, NBCA group) and absence of NBCA glue (n = 17, n-NBCA group), respectively. The operation time, blood loss, postoperative hospitalization, and liver function were compared between the two groups.

**Results:**

There was no mortality during the operation. One patient in non-NBCA group received open surgery due to parenchyma hemorrhage. Postoperative pleural effusion occurred in 2 cases of the NBCA group and 1 of the non-NBCA group. One showed left subphrenic abscess in the non-NBCA group. No postoperative bleeding occurred after 9-30 months of follow-up. The time of operation in NBCA group was significantly shorter than those in n-NBCA group (198.86±17.86 versus 217.81±20.25min, P<0.01). Blood loss in NBCA group was significantly lower than non-NBCA group (159.09±56.98 versus 212.50±88.51 ml, P<0.05). The levels of alanine aminotransferase (ALT) and aspartate aminotransferase (AST) were increased on day 1 after LSD and decreased to normal level on day 7 after LSD in both groups. There was no significant difference in postoperative hospitalization and liver function between the two groups.

**Conclusion:**

Liver retraction using NBCA glue during LSD for gastroesophageal varices and hypersplenism secondary to liver cirrhosis and portal hypertension is safe, effective, and feasible.

## 1. Introduction

Patients with chronic hepatitis B or C virus infection and chronic alcohol consumption may develop cirrhosis [1, 2], which can contribute to secondary portal hypertension and hypersplenism [3]. Gastroesophageal variceal bleeding is the leading cause of mortality in cirrhotic patients with portal hypertension [4]. In 1964, the Hassab procedure, also termed as splenectomy with azygoportal disconnection, was initially used for treating esophageal varices and hypersplenism [5]. In China mainland, Hong et al. [6] reported 20 cases of laparoscopic splenectomy and azygoportal disconnection (LSD) in 2007. Afterwards, with the advances of laparoscopic techniques, laparoscopic surgery has been commonly utilized in clinical practices. Nowadays, increasing studies have been focusing on evaluating the safety and feasibility of LSD in esophageal varices and hypersplenism secondary to cirrhosis and portal hypertension [2–4, 7–10]. Compared with open surgery, LSD can alleviate postoperative pain and contribute to the recovery of bowel function and feeding. Besides, it shows better cosmetic results and reduces the hospital stay [11]. However, due to its technical difficulties related to splenomegaly, high risk of hemorrhage, and well-developed collateral circulation, LSD has not been fully accepted by surgeons worldwide. The most important intraoperative complication during LSD is bleeding, which is the main cause of conversion [3, 11, 12]. A clear view of surgical field and suitable workspace is very important to prevent intraoperative hemorrhage. In this study, we described a method of increasing surgical exposure through liver retraction during LSD.

Liver retraction has been applied to various laparoscopic upper abdominal operations (e.g., gastrectomy, fundoplication, and hiatal hernia repair) as it contributes to the exposure of surgical fields. To date, there are several techniques for liver retraction [13–18], most of which involve a mechanical retractor and intracorporeal suturing as well as some special devices. Nevertheless, these procedures are rather complex and time-consuming. In 2014, Wu et al. [19] reported a simple and feasible liver retraction technique using n-butyl-2-cyanoacrylate (NBCA) glue in the single-incision laparoscopic upper abdominal procedures. To our best knowledge, all these liver retraction techniques are only applied to noncirrhotic patients, and rare studies have been focusing on safety and effectiveness of the technique for liver cirrhosis. In this study, the safety and efficiency of LSD in the presence or absence of NBCA glue were evaluated in 39 patients with esophageal varices and hypersplenism secondary to cirrhosis and portal hypertension in terms of operation time, blood loss, postoperative hospitalization, liver function, and major complications.

## 2. Materials and Methods

### 2.1. Study Population

Thirty-nine patients who underwent LSD between November 2014 and March 2017 in the Department of General Surgery, Qilu Hospital of Shandong University were enrolled in this study. The patients were divided into NBCA group (n=22) and non-NBCA group (n=17) based on application or no application of NBCA glue during LSD. Clinical data, including age, sex, etiology of cirrhosis, Child-Pugh stage, and spleen diameters measured by computed tomography (CT) before surgery, were recorded for each patient. Written informed consent was obtained from each patient. All operations were performed by the same surgical team using the same scheme following the surgical principles. The study protocol and procedures were approved by the Ethics Committee of Qilu Hospital, Shandong University.

### 2.2. Inclusion Criteria

The inclusion criteria were as follows: (i) those diagnosed with gastroesophageal varices and hypersplenism secondary to liver cirrhosis and portal hypertension with chronic hepatitis B or C infection or chronic alcohol consumption; (ii) those with splenomegaly confirmed by preoperative B ultrasound or CT examination; (iii) with a history of gastroesophageal variceal hemorrhage or endoscopic examination confirming severe esophageal varices, or portal hypertension and collateral circulation by CT.

### 2.3. Exclusion Criteria

Exclusion criteria were as follows: (i) those with intractable coagulation dysfunction; (ii) those showing complication with cardiopulmonary and other major organ complications, with no tolerance to general anesthesia; (iii) patients with severe perisplenitis and perisplenic adhesion; (iv) of Child-Pugh C stage; (v) intractable ascites; (vi) combined with advanced liver cancer or other malignant tumors; (vii) with a history of upper abdominal surgery and severe abdominal adhesion; (viii) unstable vital signs or hemodynamics after replenishing of blood volume.

### 2.4. Surgical Procedures

All procedures were performed under general anesthesia with the patient in the supine or semi-lateral position with a head-up tilt at 15° or with the left side elevated at 30°. The operating table could be tilted to the right or left during the surgery. Four ports were generally used, with the intra-abdominal pressure controlled at 12 mmHg.

**Figure 1:**
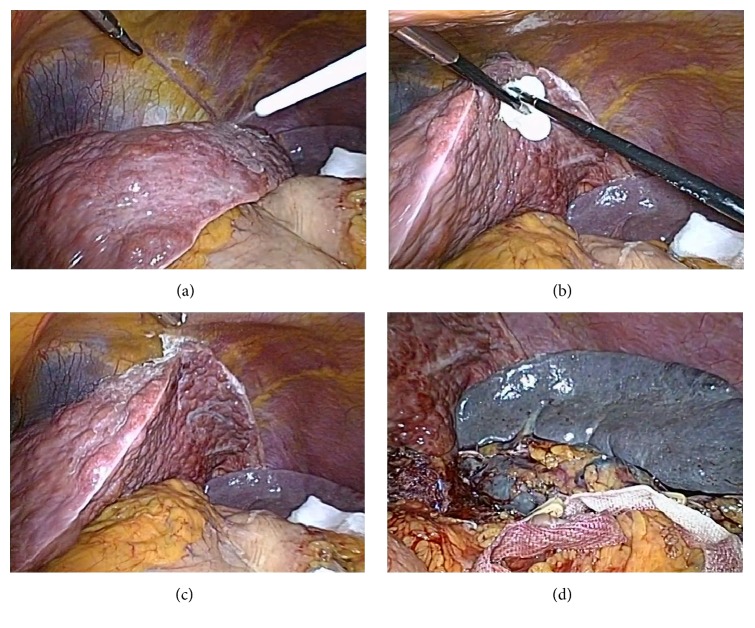
Process of liver retraction and the operation of laparoscopic splenectomy. (a) Spraying the n-butyl-2-cyanoacrylate glue on the surface of the left lateral lobe. (b) Pressing the left lateral lobe by the laparoscopic forceps against the diaphragm. (c) A clear view of surgical field and suitable workspace after liver adhesion. (d) The splenic artery and vein were transected en bloc.

For the liver retraction, the surface of the left lateral lobe was gently wiped using a surgical gauze, and then 1.5 ml NBCA glue (Compont Medical, Beijing, China) was sprayed on its surface (Figure 1(a)). The left lateral lobe was lifted up by the laparoscopic forceps enveloped surgical gauzes and pressed against the diaphragm for about 1 min to create a firm attachment to the diaphragm (Figure 1(b)). Therefore, the left lateral lobe of the liver was pasted to the diaphragm, with an aim to increase the surgical field of view and operating space (Figure 1(c)). NBCA glue would be degraded completely and absorbed after 9 months [20, 21], and there is no need to separate the liver and diaphragm after surgery.

For the procedures of laparoscopic splenectomy, the avascular area of the gastrocolic ligament was firstly opened with a harmonic scalpel (Ethicon Endo-Surgery, USA) to access the lesser sac. The splenogastric ligament including short gastric vessels, splenocolic attachments, and the splenorenal ligament were divided by using a harmonic scalpel. Whenever possible, the splenic artery was dissected and tied at the upper border of the pancreas in patients with splenomegaly [22, 23]. The splenic hilum was dissected cautiously, and the splenic artery and vein were transected en bloc with the application of a linear laparoscopic vascular stapler (Endolinear Cutter; Ethicon Endo-Surgery, USA) [3] (Figure 1(d)). The remaining spleen diaphragmatic attachments were divided using the harmonic scalpel.

**Figure 2:**
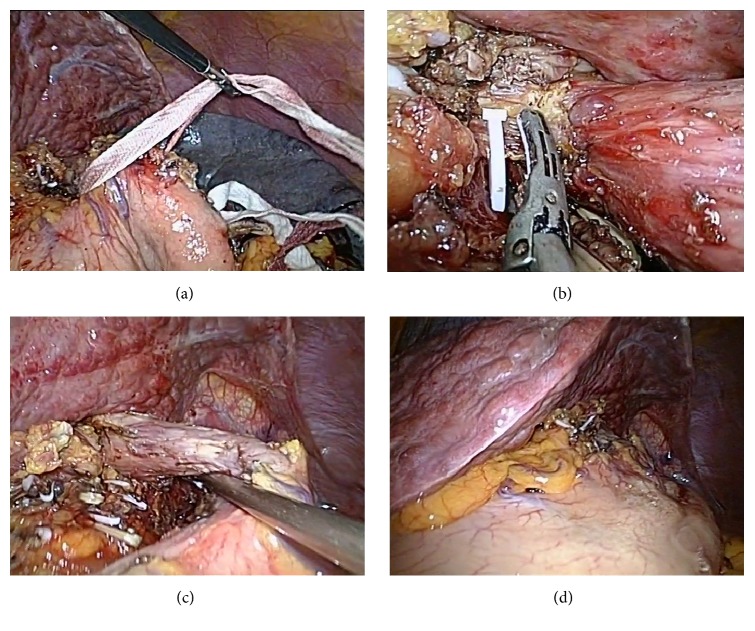
The operation of laparoscopic azygoportal disconnection and the liver adhesion lasted until the end of the operations. (a) The stomach was pulled to the left with a sterile gauze tape. (b) A surgeon was dividing the paraesophageal venous collaterals with harmonic scalpel. (c) The distal esophagus was dissected through the hiatus, and the paraesophageal venous collaterals were divided. (d) The left lateral lobe was still attached to the diaphragm when the distal esophagus devascularization finished.

In terms of laparoscopic azygoportal disconnection, the greater gastric curvature and part of the gastric fundus were divided after splenectomy, followed by dissecting upward until reaching the left crus with the harmonic scalpel and dividing of the left subphrenic vein. Subsequently, the gastrohepatic ligament was opened and then the stomach was pulled to the left with a sterile gauze tape (Figure 2(a)). The gastric coronary vein was visualized and its branches toward the esophagus and proximal stomach were divided near the esophagus and stomach walls with a harmonic scalpel (Figure 2(b)). At least, the distal esophagus (6-10 cm) was dissected through the hiatus, and the paraesophageal venous collaterals were divided [7, 12, 24, 25] (Figure 2(c)).

The liver adhesion time, duration of operation, blood loss, and major complications were recorded. Liver function tests including aspartate aminotransferase (AST) and alanine aminotransferase (ALT) were carried out before surgery and on postoperative days 1 and 7, respectively.

### 2.5. Statistical Analysis

SPSS 19.0 (IBM SPSS, USA) was used for the data analysis. All data were expressed as mean ± standard error. The difference between groups was analyzed by Student's t-test, Pearson's Chi square test, or Fisher's exact test. P<0.05 was considered to be statistically significant.

## 3. Results

There was no mortality during the operation. One patient in non-NBCA group received open surgery due to parenchyma hemorrhage. Postoperative pleural effusion occurred in 2 cases in NBCA group and 1 case in non-NBCA group. One in the non-NBCA group showed left subphrenic abscess. No postoperative rebleeding occurred after 9-30 months of follow-up. Besides, no significant differences were noticed in major clinical characteristics between the two groups (P>0.05, Table 1).

The time of operation in NBCA group showed significant decrease compared with that of non-NBCA group (198.86±17.86 min versus 217.81±20.25 min, P<0.01). Meanwhile, blood loss in NBCA group was significantly lower than that of the non-NBCA group (159.09±56.98 ml versus 212.50±88.51 ml, P<0.05). There were no significant differences in postoperative hospital stay and the platelet and leukocyte count on preoperative and postoperative day 7 between the two groups (P>0.05, Table 2).

Liver adhesion was achieved in all patients of NBCA group and lasted until the end of the operations (Figure 2(d)). The longest duration was 225 min. The liver adhesion time was 2.46±0.46 min. The levels of ALT and AST showed increase 1 day after operation and decreased to normal level on day 7 after operation in both groups. There were no significant differences in ALT or AST before surgery, 1 day or 7 days after surgery between the two groups (P>0.05, Table 3).

## 4. Discussion

NBCA glue has been commonly utilized as an adhesive for closuring the gingival flaps and mucous and cutaneous lacerations [26]. In addition, it has been used to manage the hemorrhage from the gastric varices, close fistulas, seal anastomotic leaks [27]. Besides, it was used as a method of mesh fixation for the abdominal wall during inguinal hernia repair [28]. To date, studies reported that NBCA glue showed little inflammatory and toxic effects [29]. In this study, NBCA glue was used to create adhesion between the left lateral lobe and diaphragm to obtain an adequate visualization and a suitable workspace during LSD. The time of operation was shortened to some extent, together with decreased blood loss. Moreover, no significant differences were noticed in postoperative ALT and AST between the two groups, which suggested that liver retraction using NBCA glue during LSD may not induce additional liver damage. Our data implied that liver retraction using NBCA glue was a simple, safe, and effective way to enhance exposure in LSD for esophageal varices and hypersplenism secondary to portal hypertension in cirrhosis.

The most important intraoperative complication during laparoscopic splenectomy and azygoportal disconnection is bleeding, which is the main cause of conversion [3, 11, 12]. Capsule or small vessel tears may cause oozing, which contaminates the operating field and makes the surgical procedure more difficult [3]. A clear view of surgical field and suitable workspace is very important to prevent intraoperative hemorrhage. In our study, the shortened time and decreased blood loss in NBCA group may be related to the increase of surgical exposure by using the liver retraction technique. These benefits may produce a clear view of surgical field and suitable workspace. With the enhanced exposure of surgical field, surgeon could achieve the hemostasis effectively. Besides, the assistant may undergo less labor-extensive procedures to retract liver especially during the azygoportal disconnection.

In our study, liver adhesion was achieved in all cirrhosis patients and lasted until the end of the operations, for up to 225 min. There was no necessity for additional adhesive procedures. As there might be transient elevation of aminotransferases after prolonged liver retraction [19], we also examined ALT and AST levels in NBCA and non-NBCA groups. The levels of ALT and AST increased 1 day after operation and decreased to normal level on day 7 after operation in both groups. No immediate or short-term adverse clinical effects were reported. There were no significant differences in ALT or AST between the two groups, which implied the safety of the technique.

There were limitations indeed. Firstly, we did not include the patients with lesions on the diaphragm or the dorsal surface of the left lobe of the liver (e.g., cyst and haemangioma). Secondly, as the cirrhotic liver was not smooth in texture, the liver adhesion procedure was more technical demanding than that in noncirrhotic patients. Compared with previous study [19], liver retraction in cirrhosis usually involves a longer duration [2.46 (range 1.5-3.0) versus 1.5 (range 0.8 -2.0) min]. According to our experience, the surface of the left lateral lobe should be wiped clean with a surgical gauze prior to spraying of the NBCA glue. Then the left lateral lobe of the liver was pressed against diaphragm for at least 60s to create a firm attachment. Thirdly, this study only focused on the safety and feasibility of liver retraction using NBCA glue in LSD. In future, more studies are needed in patients with cirrhosis during other laparoscopic upper abdominal surgeries, such as gastrectomy, fundoplication, and hiatal hernia repair. What is more, there are several techniques for liver retraction [13–18], most of which involve a mechanical retractor, intracorporeal suturing, and some special devices. Compared with these methods, using NBCA glue to elevate the left lateral lobe may decrease an extra incision and reduce the risk of liver tear caused by intracorporeal sutures. However, the operation time and blood loss were not compared between liver retraction using NBCA glue and other methods, and more comparative studies are needed in following studies. Finally, the sample size is small and the follow-up time is very short in this study. On this basis, a longitudinal study with longer follow-up should be performed to assess the short- and long-term outcomes in NBCA group.

In conclusion, patients in NBCA group showed shortened operation time and decreased blood loss compared with non-NBCA group. The level of ALT and AST after operation was comparable between the two groups. Our data suggest that liver retraction using NBCA glue is a simple, safe, and effective way to enhance exposure in LSD in patients with liver cirrhosis.

## Figures and Tables

**Table 1:** Clinical characteristics of the patients.

Variable	NBCA group (n=22)	Non-NBCA group (n=16)	P value
Age (yrs)	52.18±6.68	50.69±7.20	0.514
Sex			0.729
Male	15	12	
Female	7	4	
Child-Pugh class			1.000
A	13	10	
B	9	6	
Etiology			1.000
HBV+ cirrhosis	19	14	
Alcoholic cirrhosis	3	2	
spleen diameters	22.73±4.56	23.44±3.97	0.620

**Table 2:** Perioperative parameters in two groups.

Variable	NBCA group (n = 22)	Non-NBCA group (n = 16)	P value
Time of operation (min)	198.86±17.86	217.81±20.25	0.004
Blood loss (ml)	159.09±56.98	212.50±88.51	0.030
Postoperative hospital stay (day)	11.14±2.01	11.13±1.78	0.660
PLT counts (×10^9^/L)			
Preoperative	48.64±13.98	52.44±23.33	0.535
Postoperative day 7	206.09±28.90	227.81±73.25	0.213
WBC counts (×10^9^/L)			
Preoperative	3.07±0.77	3.22±1.65	0.693
Postoperative day 7	8.85±1.30	9.11±2.17	0.648
Liver adhesion time (min)	2.46±0.46		
Complications			
Pleural effusion	2	1	
Left subphrenic abscess	0	1	

PLT: platelet; WBC: leukocyte

**Table 3:** Liver functions in two groups.

Variable	NBCA group (n=22)	Non-NBCA group (n=16)	P value
ALT (unit/L)			
Preoperative	31.64±19.22	28.75±9.94	0.587
Postoperative day 1	35.32±6.12	34.50±18.22	0.845
Postoperative day 7	27.55±6.50	25.13±11.18	0.406
AST (Unit/L)			
Preoperative	36.45±18.79	33.06±11.23	0.525
Postoperative day 1	50.55±10.63	49.56±19.70	0.844
Postoperative day 7	33.05±6.00	33.13±10.55	0.977

ALT: alanine aminotransferase; AST: aspartate aminotransferase

## Data Availability

The data used to support the findings of this study are available from the corresponding author upon request.
